# A Qualitative Exploration of the Multidimensional Influences on Physical Activity Among People With Arthritis

**DOI:** 10.1002/msc.70257

**Published:** 2026-08-16

**Authors:** Justin Webb, Heather Allison

**Affiliations:** ^1^ School of Social Sciences and Professions London Metropolitan University London UK

**Keywords:** arthritis, behaviour change, physical activity, qualitative research, social ecological model

## Abstract

**Objective:**

To explore the individual, interpersonal, community, organisational, and policy‐level factors that influence physical activity among people with arthritis in England, using the Social Ecological Model as the analytical framework.

**Study Design:**

Qualitative research.

**Methods:**

Semi‐structured interviews were conducted with three stakeholder groups: people with arthritis (*n* = 10), healthcare professionals (*n* = 18), and providers of community physical activity services (*n* = 6). Participants were recruited via the charity Arthritis UK. Interviews were audio‐recorded, transcribed verbatim, and analysed thematically, with the identified themes mapped to the domains of the social‐ecological model.

**Results:**

Factors influential on physical activity were identified at individual, interpersonal, community, organisational, policy, and environmental levels. Individual‐level factors included the relationship with physical activity, knowledge, understanding and reassurance, physical and emotional pain, and alignment with personal interests. Interpersonal influences encompassed support from family and peers, and conversations with healthcare professionals, particularly physiotherapists. Community‐level factors included access, suitability, and the opportunity for social interactions. Organisational‐level factors included short‐term funding, staffing shortages and expertise, and signposting challenges. Policy and environmental factors included social norms around physical activity, and the interpretation of the physical activity guidelines.

**Conclusions:**

Physical activity promotion for people with arthritis requires a multi‐level approach that addresses barriers and harnesses facilitators across the social‐ecological spectrum. Tailored advice, improved referral pathways, sustainable funding, and inclusive activity promotion and provision are needed to support participation. These findings can inform the design of person‐centred, contextually relevant interventions to increase physical activity in this population.

## 1 Introduction

It is estimated that as many as 60% of people with arthritis are physically inactive (Pinto et al. 2017) despite physical activity being beneficial, reducing pain, improving joint function, and supporting long‐term mobility (Brady et al. 2023; Gay et al. 2016; Kessler et al. 2021). Physical activity enhances cardiovascular fitness and supports a healthy weight, which reduces mechanical load on weight‐bearing joints. Beyond physical benefits, being active improves mood, energy levels, and overall quality of life (Lopes et al. 2023). Interventions aimed at changing behaviour often focus on influencing individual factors (Bauman et al. 2012) resulting in small changes with effect sizes decreasing over time (Howlett et al. 2019). Approaches to changing physical activity should intervene not only at an individual level but also at a community and systems level (World Health Organisation 2018).

The World Health Organisation explicitly recommends that older adults should undertake regular physical activity aiming for at least 150‐min of moderate intensity or 75‐min of vigorous intensity physical activity each week. In addition, older adults should perform exercises and activities that emphasise functional balance and strength training on at least 3 days per week (World Health Organization 2020). A UK physical activity risk consensus group concluded that the benefits of being physically active outweigh the risks for people with musculoskeletal conditions and that the risk of adverse events is low. However, the consensus group states that people should start slow and build time and intensity gradually, with activity modification where necessary (Reid et al. 2022). The importance of physical activity is supported by condition‐specific guidance from the Osteoarthritis Research Society International (Holden et al. 2023) and the European Alliance of Associations for Rheumatology (Osthoff et al. 2018).

A growing body of research has explored the factors influencing physical activity among people with arthritis across multiple levels. Qualitative and mixed‐methods studies have identified a range of individual, social, and contextual barriers and facilitators, including pain, motivation, knowledge, and social support (Qvarfordt et al. 2019; Riggs and Killingback 2019). More recent research has examined the implementation of structured exercise programmes, highlighting the importance of environmental and organisational factors such as accessibility, service design, and support from healthcare professionals (Bhardwaj et al. 2024; Matile et al. 2024). Studies have also explored the perspectives of both patients and healthcare providers, demonstrating differences in perceptions of physical activity engagement and the role of clinical advice (Austin et al. 2013; Iversen et al. 2015).

While this literature provides valuable insights, much of the existing research has focused on specific programmes, conditions, or single stakeholder groups, often without integrating perspectives across multiple levels of influence. Although some studies have adopted multi‐level frameworks, there remains limited qualitative research that brings together the perspectives of people with arthritis, healthcare professionals, and community providers within a single analytic framework. As a result, little is known about how influences on physical activity interact across the social‐ecological spectrum in real‐world contexts.

Furthermore, behaviour is context‐specific, and, to the authors' knowledge, no previous research has comprehensively examined the multi‐dimensional influences on physical activity among people with arthritis within an England context. This study addresses this gap by applying the Social Ecological Model (SEM) to integrate multi‐stakeholder perspectives and provide a comprehensive, multi‐level understanding of the factors influencing physical activity in this population.

Taking a social‐ecological approach when developing an intervention strategy recognises the complex interactions between a person and the context in which they live their lives (Sallis et al. 2008; World Health Organisation 2018). Knowledge of the widespread influences on physical activity can enable the development of multilevel interventions to improve the likelihood of successful long‐term behavioural change (Bauman et al. 2012).

The SEM conceptualises behaviour as the result of dynamic interactions across multiple levels of influence, including individual, interpersonal, community, organisational, and policy environments (Sallis et al. 2008). Rather than focussing solely on individual determinants, the model recognises that behaviours such as physical activity are shaped by social relationships, environmental contexts, and broader systems (Bauman et al. 2012).

The SEM has been used to comprehensively assess physical activity within the general population (Bauman et al. 2012) but not in understanding physical activity in people with arthritis. A qualitative exploration using social‐ecological domains (Table 1) is particularly valuable as it captures the interplay between individual, interpersonal, community, organisational, and policy‐level factors that influence physical activity, providing the depth needed to uncover how these factors are experienced, perceived, and negotiated in daily life.

**TABLE 1:** Domains of the social ecological model.

Social ecological model domain	Domain description
Individual	Includes the knowledge, attitudes, past behaviours, and the characteristics of individuals such as age and gender.
Interpersonal	Includes support from family and friends, and inclusion in social networks.
Community	Includes wider community and local environment factors, for example, access to facilities.
Organisational	Includes interactions with, and the rules and regulations of, institutions, for example healthcare organisations.
Policy	Includes the influence of local, national or international policy and the macro environment on behaviour.

### 1.1 Research Aim

This research aims to qualitatively understand the individual, interpersonal, community, organisational, and policy factors that influence physical activity among people with arthritis in England.

## 2 Method

### 2.1 Study Design

This study used a qualitative design, combining semi‐structured interviews with thematic analysis to develop a deeper understanding of the factors that influence physical activity among people with arthritis. The reporting of this study adheres to the ‘Standards for Reporting Qualitative Research (O’Brien et al. 2014).

### 2.2 Participants

Participants were recruited from three stakeholder groups: (1) people with arthritis, (2) healthcare professionals involved in their care, and (3) people involved in the delivery of community physical activity opportunities. Eligibility criteria for people with arthritis included being aged 18 years or older, living in England, and having a self‐reported diagnosis of arthritis. Healthcare professionals were required to have experience supporting people with arthritis in a clinical or community setting. Physical activity providers were eligible if they were involved in the design or delivery of community‐based physical activity opportunities accessible to people with arthritis.

As part of the grant funding agreement, the research team provided an estimate of the sample size required to meet the study aims and the anticipated timeframe for data collection. This estimate was informed by the concept of Information Power (Malterud et al. 2016), which considers factors such as the study aim, sample specificity, theoretical framework, quality of dialogue, and analytic strategy.

Participants were identified and recruited by the research team in collaboration with the UK charity Arthritis UK. The charity disseminated study invitations via their networks, including mailing lists, partner organisations, and community groups. Physical activity providers were primarily identified through organisations known to deliver relevant programmes, while healthcare professionals and people with arthritis were recruited using a convenience sampling approach. Interested people were provided with an information sheet and invited to contact the research team directly to express their interest. Those meeting the inclusion criteria were enroled and provided written informed consent prior to participation. Data collection took place between February and June 2023.

The study aim, identifying the factors that influence physical activity, was considered relatively broad, suggesting the need for a larger sample. While purposive sampling ensured high relevance to the research question, particularly among the physical activity providers recruited via the charity Arthritis UK, a more heterogeneous (convenience) sample of healthcare professionals and people with arthritis was included to capture a wider range of perspectives. The use of an established theoretical framework (the SEM), alongside semi‐structured interviews, was expected to enhance the depth and quality of data, thereby increasing Information Power. However, the planned cross‐case thematic analysis supported the inclusion of a larger sample. Taking these factors into account, a total sample size of over 30 participants was considered appropriate to achieve sufficient Information Power (Braun and Clarke 2021; Malterud et al. 2016).

### 2.3 Interviews

Semi‐structured interviews were conducted by J.W. and H.A., both of whom have experience in qualitative research and public health. No additional moderators were involved. Interviews were conducted either via telephone or Microsoft Teams, according to participant preference.

Interviews were audio‐recorded with participant consent using digital recording software for the telephone interviews and video recordings for interviews that took place on MS Teams. Recordings were transcribed verbatim by a professional transcription service and checked for accuracy by the research team. Basic demographic information (e.g., age range, gender, and role) was collected prior to the interview via a short questionnaire or confirmed at the start of the interview.

An interview topic guide (see Supporting Information S1) was developed by the research team. Guides were tailored slightly for each stakeholder group while maintaining consistency in core topic areas. The guides were reviewed within the research team and refined iteratively during the early stages of data collection to improve clarity, flow, and depth of responses.

### 2.4 Data Analysis

Data were managed and analysed using Nvivo. A reflexive thematic analysis was conducted following the six‐phase approach outlined by Braun and Clarke (2006),  (2021): (1) familiarisation with the data, (2) generation of initial codes, (3) searching for themes, (4) reviewing themes, (5) defining and naming themes, and (6) producing the report.

Both researchers (J.W. and H.A.) were involved in the coding process. Transcripts were read and re‐read to support familiarisation, and initial codes were generated inductively from the data. Coding was conducted independently in the early stages, followed by regular discussions between researchers to compare interpretations, refine codes, and resolve discrepancies. Codes were then grouped into broader categories and candidate themes, which were iteratively reviewed and refined through ongoing team discussions.

Following initial theme development, a deductive approach was applied to map themes to the domains of the SEM. This combined inductive–deductive approach enabled both data‐driven insights and theoretically informed interpretation. Throughout the analytic process, themes were grounded in participant accounts, with reflexive discussions supporting credibility, consistency, and depth of interpretation. Each participant was given a pseudonym to protect their identity.

## 3 Results

Thirty‐four participants were interviewed (18 healthcare professionals of 40 invited, 10 people with arthritis of 40 invited, and 6 community physical activity service providers of 20 invited) with interviews lasting on average 30‐min (range 18–44‐min). Participant details are presented in Tables 2, 3, 4. The direct quotes included are identified by the given pseudonym and HCP for healthcare professionals, PWA for people with arthritis, and SP for community physical activity service providers.

**TABLE 2:** Healthcare professionals.

Pseudonym	Role	Gender	Age range
Tom	Physiotherapist	Male	40 to 49
Tam	Podiatrist	Female	30 to 39
Claire	Physiotherapist	Female	30 to 39
Edward	General practitioner	Male	40 to 49
Elsie	Physiotherapist	Female	40 to 49
Fiona	Occupational therapist	Female	30 to 39
Lawrence	Nurse	Male	Over 60
Maggie	Nurse	Female	20 to 30
Mabel	Occupational therapist	Female	40 to 49
Nora	Physiotherapist	Female	Over 60
Nina	Occupational therapist	Female	Over 60
Olivia	Physiotherapist	Female	50 to 59
Sam	Occupational therapist	Female	50 to 59
Stacey	Physiotherapist	Female	30 to 39
Trina	Nurse	Female	40 to 49
Sarah	Occupational therapist	Female	40 to 49
Tessa	Health and wellbeing coach within a GP surgery	Female	50 to 59
Uma	Nurse	Female	50 to 59

**TABLE 3:** People with arthritis.

Pseudonyms	Condition	Gender	Age range
Florence	Osteoarthritis	Female	60 to 69
Hilda	Osteoarthritis	Female	60 to 69
Maya	Inflammatory arthritis	Female	50 to 59
Nancy	Gout, osteoarthritis	Female	60 to 69
Ruby	Osteoarthritis	Female	70 to 79
Sadie	Inflammatory arthritis	Female	50 to 59
Thea	Osteoarthritis	Female	80 to 89
Tiffany	Inflammatory arthritis	Female	60 to 69
Ursula	Osteoarthritis	Female	60 to 69
Nicole	Osteoarthritis	Female	60 to 69

**TABLE 4:** Community physical activity service providers.

Pseudonym	Role	Gender	Age range
Barry	Director of a community interest company	Male	40 to 49
Trevor	Coach/instructor	Male	30 to 39
Steve	Sports development worker	Male	40 to 49
Olive	Coach/instructor	Female	30 to 39
Thomas	Club director and coach/instructor	Male	50 to 59
Orla	Clinical service lead and physical activity programme co‐ordinator	Female	30 to 39

### 3.1 Themes

Thematic analysis was conducted across the combined dataset, integrating perspectives from people with arthritis, healthcare professionals, and community physical activity service providers. Data from all three stakeholder groups were coded and analysed collectively, allowing for the identification of shared patterns as well as points of convergence and divergence across experiences. Codes were grouped into categories and developed into themes, which were subsequently organised according to the domains of the SEM.

Each theme represents a distinct but interconnected influence on physical activity, reflecting how individual experiences are shaped by interpersonal relationships, community contexts, organisational structures, and wider policy environments. The themes are presented with illustrative quotes to highlight their meaning in relation to physical activity behaviour (additional quotes for each theme are available in Supporting Information S2).

#### 3.1.1 Individual Factors That Influence Physical Activity

##### 3.1.1.1 Relationship With Physical Activity—Closer to Personal Identity to Complete Disengagement

Participants expressed varied relationships with physical activity, ranging from strong personal identification to complete disengagement. For some, being active was integral to their identity and daily life; they adapted to arthritis by modifying activities rather than abandoning them: ‘If I can't swim, then I'll do aqua aerobics.’ (Nancy—PWA);

‘It is part of my routine. I always have a walk… every day… even if I don't feel like it.’ (Hilda—PWA). Others described how their activity declined following diagnosis, accompanied by frustration and a sense of loss: ‘I was really active… but subsequently I haven't been able to do that.’ (Sam—PWA).

Some viewed physical activity as unappealing or irrelevant. Community physical activity service providers and healthcare professionals echoed this, noting resistance and scepticism towards exercise: ‘There's a reluctance on lots of people's parts to do any exercise, having some pieces of equipment which are quite a giggle… does help… it's quite good to have a whole range of activities so people can dip in and dip out.’ (Orla—SP). Healthcare professionals suggested that some believed that they could not be active because they perceived themselves as not able. Others experienced benefits that encouraged activity: ‘I think lots of individuals notice themselves the benefits.… it's helped them physically and mentally.’ (Thomas—SP).

These perspectives reflect a continuum, from those who see physical activity as part of who they are, to those who no longer feel capable or interested, highlighting the importance of recognising differing identities, motivations, and beliefs in promoting activity.

##### 3.1.1.2 Knowledge, Understanding, and Reassurance

Knowledge about the importance of physical activity varied. While some healthcare professionals believed the benefits were common knowledge, others identified misconceptions and a lack of understanding about physical activity: ‘There's a lack of understanding of the role that exercise has and how important it is…. it's… useful to empower people to know what is safe.’ (Tam—HCP). Patients sought reassurance: ‘I've Googled so much you wouldn't believe. I went searching for reassurance.’ (Florence—PWA).

##### 3.1.1.3 Physical and Emotional Pain

Pain was both a physical and emotional barrier, creating feelings of grief and loss: ‘They've lost their mobility…independence. It's always hard dealing with loss and I think it does bring up a lot of emotion in patients.’ (Tam—HCP). Some showed resilience in the face of this: ‘You just had to grit your teeth and go’ (Ruby—PWA). Negative experiences, such as pain after activity, reinforced inactivity: ‘The more I walk, the more I stand, the worse the pain gets.’ (Thea—PWA).

##### 3.1.1.4 Activity as a Pathway to What Matters

‘Taking into account what is important to the patient’ (Stacey—HCP) is essential when considering how to encourage people with arthritis to move more. For some, being active was about maintaining independence, or being outside in nature, being able to socialise, or play with their kids and grandkids. Music seemed important in many people's lives and a reason to be active through dancing: ‘Friday we've got a musician… she [person with arthritis] gets up and dances every Friday. She doesn't do the exercise [classes]… but she always dances.’ (Orla—SP).

#### 3.1.2 Interpersonal Factors That Influence Physical Activity

##### 3.1.2.1 The Social Dimension of Physical Activity

Family, friends, and peers played a powerful role in shaping attitudes. For some, support and shared routines encouraged participation: ‘My partner… likes to keep healthy…. we try and encourage each other.’ (Sam—PWA). However, well‐meaning friends and family sometimes discouraged activity due to safety concerns: ‘[Friends say] Oh, I wouldn't run, [you've] got a bad knee.’ (Florence—PWA). Social networks outside of the family were also influential. Many participants valued the companionship and sense of belonging fostered through group activities: ‘I also go to Slimming World on a Friday, and we've got a lovely little support group there… we go for coffee after… have a cry sometimes and have a laugh sometimes… just sharing, being part of a community.’ (Ruby—PWA).

Overall, social relationships acted as both facilitators and barriers to physical activity. Supportive partners and peers fostered motivation and accountability, whereas protective or discouraging attitudes from others could undermine confidence and participation.

#### 3.1.3 Conversations About Physical Activity—Brief, Variable, and Skill‐Dependent

Healthcare professionals reported that physical activity was discussed with most patients briefly and informally. For some, this advice was impactful and enduring:

‘The orthopaedic surgeon said to me… walk every day if you can. I always remember that.’ (Hilda—PWA). However, limited consultation time often constrained the depth of these discussions: ‘With health professionals you feel you can't really talk to them too much in depth because they've got a tight schedule.’ (Sadie—PWA).

Allied health professionals highlighted that the nature and extent of advice varied between roles and individual clinicians: ‘The consultant I work with is quite reticent about suggesting that people could run… fear of bone health and fractures.’ (Olivia—HCP). Several healthcare professionals noted that effective conversations relied on communication skills such as motivational interviewing and gentle ‘nudging,’ rather than directive advice: ‘Very quickly patients will say, ‘I've tried that…’ You've got to have the confidence and the commitment.’ (Tom—HCP). Physiotherapists were considered experts: ‘I'm nowhere near an expert such as like a physio.’ (Uma—HCP).

#### 3.1.4 Community Factors That Influence Physical Activity

##### 3.1.4.1 Access, Availability, and Suitability of Community Activities

People with arthritis and healthcare professionals described a range of community‐based opportunities (e.g., walking groups, yoga, aqua aerobics, boccia, and dance). While choice was broad, barriers such as transport, timing, cost, and the suitability of sessions for varying ability levels limited participation. Small adaptations, such as slower pacing, alternative movements, and accessible venues, were seen as key to inclusion: ‘It needs consideration for some less able people who are really willing… they just need adapted moves.’ (Uma—HCP).

##### 3.1.4.2 Community Programmes as Social Spaces

Beyond physical benefits, community activities provided valuable opportunities for social connection. Community physical activity service providers emphasised intentionally fostering interactions, pairing participants, encouraging post‐session gatherings, and creating welcoming environments that nurtured belonging: ‘We put people into pairs… as soon as they've finished a session they'll then head off and have tea, cake, coffee, etc.’ (Thomas—SP).

#### 3.1.5 Organisational Factors That Influence Physical Activity

##### 3.1.5.1 Short‐Termism and Funding Instability

Short‐term fragmented funding models are a persistent challenge across community settings. Programmes were often funded for limited periods (typically 6–12 weeks), restricting the ability to build habits or sustain delivery: ‘Six weeks isn't really long enough to create a habit of exercise.’ (Claire—HCP).

Community physical activity service providers described relying on multiple small grants or delivering sessions at a loss, creating uncertainty and administrative burden: ‘We actually run it at a loss and have been doing for 16‐months’ (Orla—SP). Although some organisations absorbed costs for participants facing financial barriers, this was not sustainable. Funding instability undermined long‐term planning, workforce retention, and the consistency needed to support people with arthritis in maintaining physical activity.

##### 3.1.5.2 Workforce Capacity and Expertise

Staffing shortages across healthcare and community settings, particularly physiotherapists, occupational therapists, and trained exercise professionals, were seen as major barriers: ‘There's a real shortage of people who can talk to patients, who can have those difficult conversations.’ (Tam—HCP).

Access to appropriately skilled staff within General Practice and community services was identified as a facilitator, enabling needs assessments and ongoing support. However, delivering person‐centred approaches at scale was challenging: ‘It becomes much more challenging to try and formulate some type of personalised but at the same time broadly implementable type [of] programme.’ (Olivia—HCP).

##### 3.1.5.3 Systems for Signposting and Referral

Healthcare professionals reported difficulty keeping up‐to‐date with local activity options, which limited their ability to refer patients effectively. Centralised directories were helpful where available: ‘We've got a website… that's got all the activities and exercise classes in the whole of [location]… really useful.’ (Elsie—HCP).

Quality assurance concerns, information governance, and complex referral processes further hindered access: ‘I would not… [signpost] unless I was aware of what that service provision was and who was running it.’ (Uma—HCP); ‘My physio recommended hydrotherapy… I never received the form.’ (Sonia—PWA). Follow‐up to ensure patients accessed community activities was rare due to resource limitations and workload pressures.

#### 3.1.6 Policy and Environmental Factors That Influence Physical Activity

##### 3.1.6.1 Norms and Representation

Cultural and social norms influenced perceptions of who physical activity was ‘for.’ Healthcare professionals described how stereotypes and self‐perceptions discouraged participation, particularly among people who felt they did not fit the image of someone who exercises.

Inclusive imagery and messaging were seen as effective in challenging these assumptions and promoting participation. Visual resources that depicted older adults and people with arthritis being active were said to ‘normalise’ physical activity and make it appear achievable: ‘They're elderly people who are obese, who've clearly got arthritis and are exercising, so that instantly normalises this idea.’ (Edward—HCP). This highlights the importance of representation in shaping social norms and creating environments where diverse bodies and abilities are visibly included in the narrative of being active.

##### 3.1.6.2 Interpreting National Guidelines

Healthcare professionals valued national guidelines but expressed concern that they were often unrealistic. Recommendations such as 150‐min of activity per‐week were viewed as unattainable for many, risking discouragement rather than motivation: ‘The national guideline is 150‐min a week… If the only exercise you get is walking from your armchair to the loo… it's meaningless to say you should be getting 150‐min a week.’ (Edward—HCP).

Healthcare professionals emphasised the need to translate guidelines into tailored, flexible advice that recognised peoples' lived experiences. Framing guidance in terms of what is possible rather than what is prescribed was seen as important for sustained engagement.

A summary of the themes by social‐ecological domain is presented in Table 5.

**TABLE 5:** Themes by social‐ecological domain.

Social‐ecological domain	Themes
Individual level	Relationship with physical activity—closer to personal identity to complete disengagement
	Knowledge, understanding, and reassurance
	Physical and emotional pain
	Activity as a pathway to what matters
Interpersonal level	The social dimension of physical activity
	Conversations about physical activity—brief, variable, and skill‐dependent
Community level	Access, availability, and suitability of community activities
	Community programmes as social spaces
Organisational level	Short‐termism and funding instability
	Workforce capacity and expertise
	Systems for signposting and referral
Policy and environmental level	Norms and representation
	Interpreting national guidelines

## 4 Discussion

This paper aims to qualitatively understand the multidimensional factors influencing the physical activity of people with arthritis. Although the findings are presented according to the domains of the Social Ecological Model, the influences on physical activity were often interconnected and could reasonably be interpreted across more than one domain.

The categorisation of themes was intended as an analytical framework to aid interpretation, rather than to imply that influences operate independently of one another. Themes were mapped according to the primary mechanism through which they influenced physical activity from the perspective of the patient with arthritis. For example, social support extends beyond interpersonal relationships into community settings. This interconnectedness reflects the principles of the Social Ecological Model, which recognises that behaviour is shaped through dynamic interactions across multiple levels of influence.

This study highlights the need for multi‐level approaches to improving physical activity, recognising that barriers and facilitators extend well beyond individual motivation (World Health Organisation 2018).

### 4.1 Implications for Practice

Health professionals, particularly physiotherapists, are well placed to provide personalised advice, reassurance on safety, and referral to appropriate physical activities. Advice from healthcare professionals has been shown to increase physical activity (Sarısaltık et al. 2023). However, such conversations require time, skill, and confidence, suggesting a need for training in motivational interviewing and behaviour change (Vishnubala et al. 2022).

The guidelines for physical activity of 150‐min per week of moderate intensity activity are often out of reach. Health benefits can be achieved with less (Ginis et al. 2021). The importance and benefits of physical activity for general health and to support condition self‐management need to be communicated to people with arthritis in inclusive and accessible formats (Holden et al. 2023; Osthoff et al. 2018; World Health Organization 2020). Communication materials should reflect diverse body types, abilities, and ages to challenge social norms that discourage participation.

Well‐meaning family and friends often discourage activity. However, partners can be a catalyst for moving more (Kanavaki et al. 2017; Richards et al. 2018). It may be necessary to engage those closest to a person with arthritis in discussions about the importance of being active. Those that see physical activity as part of condition self‐management, experiencing the benefits, are likely to continue to be active if they have the tools to do so safely and correctly (Kanavaki et al. 2017). Discussing the personal positive consequences of increased physical activity, and incorporating movement in to pursuits already enjoyed by people with arthritis, can support sustained change (Brady et al. 2023; Kwasnicka et al. 2016). The setting of personal goals based on preferences can increase motivation and commitment to change (Kwasnicka et al. 2016).

Condition‐specific physical activity guidelines for people with arthritis, including those from the Osteoarthritis Research Society International (Holden et al. 2023) and the European Alliance of Associations for Rheumatology (Osthoff et al. 2018) emphasise the importance of knowledge, understanding, reassurance, and the role of exercise within arthritis self‐care and self‐management. Condition‐specific guidance also recognises the responsibility of healthcare professionals to discuss physical activity with patients; however, less emphasis is placed on the social dimensions of physical activity, opportunities within community settings, referral pathways, and workforce capacity and expertise. In contrast, community‐based physical activity is more explicitly recognised within the World Health Organisation guidelines for physical activity (2020). Findings from the present study suggest that stronger links between healthcare and community physical activity provision are needed to give healthcare professionals greater confidence in the quality, appropriateness, and safety of available opportunities.

The inclusion of community providers in this research helped illuminate the important role of community settings in supporting physical activity through adaptation, social connection, and ongoing participation. Referral pathways from healthcare to the community need to be improved, supported by up‐to‐date quality‐assured directories of provision. Social prescribing has the potential to bridge the current gap. Social prescribing aligns with a person‐centred approach, recognising the social, emotional, and practical influences on physical activity behaviour, and providing tailored support taking into account people's preferences and motivations (Jacobsen et al. 2025). Social prescribing can relieve some of the burden on clinicians, who often report a lack of time, resources, and confidence in signposting. It can also promote continuity and sustainability by fostering stronger partnerships between healthcare and community organisations.

Small adaptations to community activities such as pacing adjustments, alternative exercises, or equipment changes can make participation more feasible. Social influences, reinforcement, and support in managing physical activity behaviour change in the long‐term are important motivators in older adults (Spiteri et al. 2019). Embedding social elements within physical activity sessions may improve retention by meeting participants' interpersonal needs.

Policy‐level influences were less frequently articulated by participants, which may reflect how distal policy drivers are often experienced indirectly through services, funding arrangements, public messaging, and local opportunities rather than recognised as policy in everyday life. Nevertheless, the findings suggest that policy remains important, particularly in relation to national physical activity guidance, commissioning priorities, short‐term funding models, and support for community‐based provision. This highlights the need for policy approaches that create enabling environments for physical activity, rather than relying solely on individual behaviour change. Sustainable funding models are essential to maintain accessible and affordable opportunities beyond short‐term projects. This may involve collaboration between healthcare providers, local authorities, and voluntary sector organisations to ensure continuity.

### 4.2 Implications for Research

Future research should explore how tailored physical activity interventions, co‐designed with people with arthritis, can address barriers across all social‐ecological domains. Implementation studies are needed to test strategies for improving the transition from healthcare to community provision, including the role of social prescribing, integrated care pathways, and digital signposting tools.

Further investigation is warranted into the effectiveness of low‐cost, small‐scale adaptations in community settings, particularly in relation to participation and long‐term adherence. Longitudinal qualitative studies could provide insights into how physical activity behaviours evolve over time, especially in response to changes in pain, mobility, or social circumstances.

Given the influence of family and peers, interventions that actively engage social networks should be investigated. Research should assess the impact of inclusive imagery and messaging on participation among those who feel they do not fit the perceived profile of someone who exercises. Lastly, economic evaluations could inform funding bodies of the cost‐effectiveness of sustained, community‐based programmes compared with short‐term initiatives.

### 4.3 Strengths and Limitation of This Study

A strength of this study is the inclusion of perspectives from multiple stakeholder groups, allowing for a comprehensive exploration of the multi‐level factors that influence physical activity. The use of the SEM provides a structured lens through which to interpret findings across individual, interpersonal, community, organisational, and policy domains. The qualitative design enabled rich, detailed accounts that captured the complexity of lived experience and service provision. A key strength of this study was the inclusion of community physical activity providers alongside people with arthritis and healthcare professionals. This broadened the analysis beyond patient and clinical perspectives, enabling insight into the community setting as an important context for physical activity participation.

Limitations are acknowledged. Recruitment relied on voluntary participation, which may have attracted people already more engaged with physical activity or with stronger opinions about it, potentially limiting the representativeness of views. The sample of people with arthritis was all female and older, likely reflecting the demographic profile of people engaging with the charity Arthritis UK, limiting the transferability to younger or more male populations. Most healthcare professional participants were allied healthcare professionals, that is, qualified registered clinicians distinct from doctors, nurses, and dentists, who provide specialised diagnostic, technical, and therapeutic care (England, n.d.). There was limited representation from General Practitioners or Consultants. This may have influenced the types of conversations and barriers reported. Further, one interview with a healthcare professional was very short, at only 18‐min, as the participant had to end the interview early due to other commitments, limiting their contribution.

The experiences described may have been shaped by local service configurations and resources, which can vary regionally. It is acknowledged that the views of local decision makers, such as local authority representation, are missing from this research. Furthermore, whilst policy‐level influences were explored through the perspectives of participants, the inclusion of policy makers may have provided a more comprehensive understanding of the structural and policy factors influencing physical activity among people with arthritis.

Although the overall sample size was considered sufficient to achieve Information Power (Malterud et al. 2016), the final numbers recruited within each stakeholder group were modest relative to invitations issued, particularly among community physical activity service providers. This may have limited the range of perspectives captured within some groups and raised the possibility of self‐selection bias, whereby those more engaged with physical activity or musculoskeletal care were more likely to participate.

Finally, as this study was conducted in England, the findings are situated within a specific healthcare, policy, and community context. The organisation of healthcare services, referral pathways, and community physical activity provision varies internationally, which may influence the applicability of these findings to other countries.

## 5 Conclusion

An understanding of the system in which physical inactivity occurs is vital in bringing about change. The importance of behaviour change interventions for people and groups, the importance of advice and guidance from healthcare professionals, and the support of family and friends in improving physical activity in the general population and in those with long‐term conditions, is well documented. What is less well known, and highlighted in this research, is how small adaptations to community provision could make physical activity more accessible and welcoming for people with arthritis.

This research sheds light on the barriers that exist between healthcare and community physical activity provision, which prevents people with arthritis, and it is assumed other musculoskeletal conditions from being successfully referred to appropriate activities. Further, this research highlights the impact of short‐term funding arrangements on the ability of service providers to provide ongoing accessible opportunities for people with arthritis to be physically active.

## Author Contributions

J.W. prepared the manuscript. J.W. and H.A. completed the interviews and data analysis. J.W. and H.A. have professional and academic experience in public health and physical activity. J.W. is an Associate Professor of Physical Activity and Health with a background in behaviour change, intervention development, public health and applied research. H.A. is a Senior Lecturer and researcher with expertise in qualitative methods and musculoskeletal health.

Both authors have training and experience in qualitative research, including the design and conduct of semi‐structured interviews and thematic analysis. Their professional backgrounds informed the development of the interview topic guides, data collection, and the analytic approach. The researchers' professional perspectives may have shaped the interpretation of findings, particularly in relation to healthcare and community practice. To minimise bias, both researchers were actively involved in coding and engaged in regular reflexive discussions throughout the analytic process. Interpretations were grounded in participant accounts to enhance credibility and ensure that findings reflected participants' experiences.

## Funding

The research was funded by the charity Arthritis UK.

## Ethics Statement

Ethical approval for this research was granted by the School of Social Sciences and Professions ethics committee at London Metropolitan University in February 2022.

## Consent

All participants provided informed consent to participate and for their data to be used in publication of this research.

## Conflicts of Interest

This research was funded by the charity Arthritis UK. Arthritis UK supported the recruitment of participants to this study. No other conflicts of interest are declared by the authors.

## Supporting information


Supporting Information S1

Supporting Information S2

## Data Availability

The interview transcripts are not publicly available due to the terms of participant consent. Participants were informed that their anonymised data may be used in future studies conducted by the research team and subject to relevant ethical, legal, and regulatory approvals. Data cannot be shared with researchers external to the study team. Additional quotes in support of the identified themes are presented in Supporting Information S2.
