# Supplementary material for: A Qualitative Exploration of the Multidimensional Influences on Physical Activity Among People With Arthritis
Source: Musculoskeletal Care. 2026 Aug 16;24(3):e70257. doi: 10.1002/msc.70257 (PMC13478348; doi:10.1002/msc.70257)
Supplement: Supplementary file 1 [file MSC-24-e70257-s001.docx]

INTERVIEW GUIDE – People with arthritis

The interviews will be:-

- Semi structured
- Clear, asking simple, easy, short questions with no jargon
- Gentle, and give people time to think
- Empathetic and sympathetic
- Open and flexible responding to what is important to interviewee
- Inquisitive, clarifying statements without imposing meaning on them
- Balanced
- Sensitive.

**Structure**

These questions are to be used to lead the discussion. They do not have to be rigidly followed and are for guiding purposed only. The interviewer is to use his or her judgement to ensure that information is gleamed from each topic area.

**Introduction:**

- Name and role
- Thank participant for their time
- Inform that the interview is being recorded and explain how the data will be used
- Briefly explain the study aims and outcomes
- Confirm consent, read the information letter if necessary
- Ensure that participant is happy to continue.

**Current behaviour**: Can you tell me about how active you are at the moment?

**Past behaviour**: What about in the past?

**Services:** Have you used and physical activity services/programmes?

**Motivation**: What motivates you to be active? Why/Why not? Is activity something you feel you should do? Why? Is it something you want to do? Why? What helps you/might help you become active?

**Social influences**: Do your friends and family influence your activity? Have you ever had a conversation about activity with a healthcare professional? What professional? What did they tell you? How did you feel about the conversation?

**Knowledge**: What do you understand about physical activity/exercise for people with arthritis? Do you think it is important?

**Skills**: Do you know what to do? Do you think you are doing it correctly? Are there particular skills necessary to be active?

**Memory, attention, decision processes**: Is physical activity something that you usually do? Is it a habit?

**Behavioural regulation**: Is physical activity something that you keep track of?

**Social/Professional role and identity**: Do you describe yourself as being active?

**Beliefs about capabilities**: How easy do you find trying to stay active?

**Optimism**: How confident are you about being activity in the future?

**Beliefs about consequences**: What do you think will happen if you are more active? Is it sustainable?

**Intensions**: Do you intend to be more active?

**Goals**: How much do you want/feel that you should do more activity?

**Reinforcement**: Are there incentives to becoming more activity?

**Emotion**: Are there any emotional elements to physical activity for you? Fear, anxiety, stress, happiness, etc.

**Environmental context and resources**: To what extent does the environment, weather, facilities, other resources, help or hinder activity?

**Conclusion**:

- Is there anything that you would like to add or talk about that you think is of relevance to this study?
- Thank participant for their time and inform them of the next steps.

INTERVIEW GUIDE – Healthcare Professionals

The interviews will be:-

- Semi structured
- Clear, asking simple, easy, short questions with no jargon
- Gentle, and give people time to think
- Empathetic and sympathetic
- Open and flexible responding to what is important to interviewee
- Inquisitive, clarifying statements without imposing meaning on them
- Balanced
- Sensitive.

**Structure**

These questions are to be used to lead the discussion. They do not have to be rigidly followed and are for guiding purposed only. The interviewer is to use his or her judgement to ensure that information is gleamed from each topic area.

**Introduction:**

- Name and role
- Thank participant for their time
- Inform that the interview is being recorded and explain how the data will be used
- Briefly explain the feasibility study aims and outcomes
- Confirm consent, read the information letter if necessary
- Ensure that participant is happy to continue.

**Current behaviour:** How active are you patients?

**Past behaviour:** Has this changed?

**Knowledge:** What do you know about physical activity for people with arthritis? What do people with arthritis know about physical activity?

**Skills:** Do you know how to discuss/give advice on physical activity? What skills do people with arthritis need to be active?

**Motivation:** What motivates people with arthritis to be active?

**Services:** What services are available to them to support physical activity?

**Memory, attention, decision processes:** Is taking about activity something that you usually do?

**Behavioural regulation:** How do you keep track of these conversations? Do you remember to talk to everyone or is it just selected service users?

**Social/Professional role and identity:** Are discussion on physical activity compatible or in conflict with your professional role? What about your social identity? Do others have these conversations? What are they like? What do they entail?

**Beliefs about capabilities:** How easy or difficult is it for you? What are the problems with discussing physical activity? How capable are people with arthritis in regards to physical activity?

**Optimism:** How confident are you that any problems with discussing physical activity with service users can be overcome, personally and organisationally? How optimistic are people with arthritis about physical activity?

**Beliefs about consequences:** What do you think will happen if you discuss physical activity with service users? What do people with arthritis think will happen if they do more activity?

**Intensions:** Is discussing physical activity a conscious decision? How did you make that decision? Is this something you intent to do/continue doing/ stop doing?

**Goals:** Do you discuss physical activity enough? How much do you want/feel that you should do it?

**Reinforcement:** Are there incentives to discussing physical activity with service users?

**Emotion:** Does discussing physical activity evoke any sort of emotional response in you or your patients?

**Environmental context and resources:** To what extent do having, or not, physical resources facilitate discussions on physical activity? Does the environment/setting that you work in help or hinder such discussions? Do environmental factors influence people with arthritis becoming active?

**Social influences:** To what extent do social influences facilitate or hinder discussions on physical activity? To what extent do social influences facilitate or hinder physical activity in people with arthritis?

**Conclusion:**

- Is there anything that you would like to add or talk about that you think is of relevance to this study?
- Thank participant for their time and inform them of the next steps.

INTERVIEW GUIDE – Physical Activity Service Providers

The interviews will be:-

- Semi structured
- Clear, asking simple, easy, short questions with no jargon
- Gentle, and give people time to think
- Empathetic and sympathetic
- Open and flexible responding to what is important to interviewee
- Inquisitive, clarifying statements without imposing meaning on them
- Balanced
- Sensitive.

**Structure**

These questions are to be used to lead the discussion. They do not have to be rigidly followed and are for guiding purposed only. The interviewer is to use his or her judgement to ensure that information is gleamed from each topic area.

**Introduction:**

- Name and role
- Thank participant for their time
- Inform that the interview is being recorded and explain how the data will be used
- Briefly explain the feasibility study aims and outcomes
- Confirm consent, read the information letter if necessary
- Ensure that participant is happy to continue.

**Process:**

- Tell me about your service
- How do people with arthritis…
  - Hear about your service?
  - Access you service?
  - Interact with your service?
- How long do people with arthritis stay with your service? Where do they go afterwards? How do you motivate participants?
- Tell me about the assessment of your service users. When does this take place (if it does take place)?
- Do you feel that your service users are representative of the population of people with arthritis?
- Tell me about the activities that people with arthritis take part in.
- Is the service tailored to the service users in anyway? Is everyone treated the same?
- What is the frequency, intensity, time, type based on?
- Are there links to other services? Partnership working.

**Structure:**

- What resources are required to run your service?
- Do you have all of the necessary resources required to delivery high quality care/provision?
  - People
  - Equipment
  - Physical/virtual space
  - Time
  - Money
- Are there any user access concerns or areas for improvement?
- Tell me about the organisation that hosts the service. What are its characteristics, culture etc?

**Outcome:**

- What outcomes do you see in service users (consider physical activity measures – cardiovascular/strength; health related quality of life; retention rates, drop out rates, any others)?
  - Objective
  - Subjective
  - Group or individual
- Do you see any unexpected outcomes?
- What are the outcomes you experience as a service deliverer?
- What can you say about the capability, opportunities, and motivation of people with arthritis in regards to physical activity?

**Conclusion:**

- Is there anything that you would like to add or talk about that you think is of relevance to this study?
- Thank participant for their time and inform them of the next steps.
