# Supplementary material for: A Qualitative Exploration of the Multidimensional Influences on Physical Activity Among People With Arthritis
Source: Musculoskeletal Care. 2026 Aug 16;24(3):e70257. doi: 10.1002/msc.70257 (PMC13478348; doi:10.1002/msc.70257)
Supplement: Supplementary file 2 [file MSC-24-e70257-s002.docx]

Supplementary Table: Additional quotes in support of the identified themes

| **Social ecological domain** | **Theme** | **Selected supporting quotes** |
| --- | --- | --- |
| Individual influences | Relationship with physical activity – from identity to disengagement | “I do get some people who are quite on it and they’re very active.“ (Fiona - HCP).  “I’d say a lot of people talk about how they used to be active.” (Mabel - HCP).  “Actually, [they feel] if they’ve got this arthritis then actually, they need this medicine, they need this injection, they need this pill,” so it’s how you compete with that.” (Tom - HCP).  “I would try and go to gyms occasionally, but I never really got back the enthusiasm for it.” (Thea - PWA).  “[exercise is] boring.” (Nicole – PWA).  “Very often the reason people don’t exercise is because they feel like they can’t.” (Tam - HCP). |
|  | Knowledge, understanding, and reassurance | “It’s fairly common knowledge that to keep your body healthy you need to keep moving.” (Mabel - HCP). |
|  | Physical and emotional pain | “It’s got to the point now where I am struggling with my mood badly.” (Maya - PWA). |
|  | Activity as a pathway to what matters | “I don’t drive, so I have to walk to places at the moment anyway.” (Nicole - PWA).  “I love moving to music.” (Maya - PWA).  “For some people it might be social groups that’s the first step, that’s the important thing. It’s not physical activity but it’s an important activity to get them out of the house and engaged in society.” (Sam - HCP). |
| Interpersonal influences | The social dimension of physical activity | “They’re people that are very motivated anyway, and I’d say that a lot of those people are couples so maybe they encourage each other to do that.” (Mabel - HCP).  “Maybe it’s just having a bit more contact with people who understand how you feel and will help you through some of the difficult bits. (Tiffany – PWA). |
|  | Conversations about physical activity – brief, variable, and skill-dependent | “Weight, exercise and independence, those three things together are probably…the biggest conversations that I have with my patients.” (Trina - HCP).  “Physio said, “Try this”, and she taped me up and showed me what to do, and then I found it was brilliant. That was years ago now, about seven years ago, and ever since.” (Nancy – PWA). |
| Community influences | Access, availability, and suitability of community activities | “I like walking and I want to get back to doing at least a couple of walks a week, and along with my aqua aerobics, that should keep me going.” (Nancy – PWA).  “…gardening in the afternoon, we’ve got an allotment…We’ve done a lot of chair yoga…,chair aerobics, not everybody sits down. We’ve just bought these hula hoop things…We’ve got boccia.” (Olive – SP).  “When I ask them, it tends to be transport is an issue, getting to places.” (Elsie - HCP).  “They souped it up [the local gym] and it became one of the most intimidating experiences. I didn’t want to be there.”(Ruby - PWA).  “We did have a hydrotherapy pool at one of the hospitals. That’s been closed down” (Ruby - PWA).”  “There’s not really a lot around…similar age and similar mobility..I would like that but there’s not a lot available.” (Ursula - PWA).  “We had to move it to the village hall and the numbers just seemed to increase.” (Trevor – SP). |
|  | Community programmes as social spaces | “For some people it might be social groups that’s the first step, that’s the important thing. It’s not physical activity but it’s an important activity to get them out of the house and engaged in society.” (Sam - HCP). |
| Organisational influences | Short-termism and funding instability | “My view is they need much more flexible pathways. I think the very traditional model of, ‘You’ve got to do this for this number of weeks and then that’s it, you’ve had it, you can’t come back,’ come on!” (Olivia - HCP).  “We provide free sessions for them if we feel that there’s a barrier there…so that’s been helpful in applying for that grant.” (Trevor – SP).  “They [the funders] wanted all these different things measured at the start, at the end and it’s just not practical to be honest.” (Orla - SP). |
|  | **Workforce capacity and expertise** | “[Hydrotherapy] The sessions are really short because the service is so sought after and they’re so short of staff.” (Uma - HCP).  “[The] physio waiting list in their area of about 25/26 weeks.” (Steve – SP). |
|  | **Systems for signposting and referral** | “Once we’ve got them to a point where it’s only a case of continuing self-management, it would be lovely if there was a local group that I could refer them to, to help give them that community feeling to be able to continue their exercise within.” (Stacey - HCP).  “But information governance makes a massive barrier for voluntary sector to be part of the NHS unfortunately.” (Orla - SP). |
| Policy and environmental influences | Norms and representation | “I think maybe posters, some sort of, something within GP practices or within healthcare settings that empower people to be more active. I think that would be good, eye-catching slogans and that sort of thing, even TV adverts, that sort of thing would work well. Because I think we just need to get it into the mass consciousness, or even sub-consciousness that actually people can move and should be more.” (Tom – HCP). |
|  | Interpreting national guidelines | “150 minutes per week, what is that based on? A healthy person who doesn’t live with pain, who can move their joints.” (Nora -HCP) |
